# Elevated serum triglycerides is the strongest single indicator for the presence of metabolic syndrome in patients with type 2 diabetes

**DOI:** 10.1186/1475-2840-5-21

**Published:** 2006-10-04

**Authors:** Maria Kompoti, Anargiros Mariolis, Alevizos Alevizos, Ioannis Kyrazis, Ioannis Protopsaltis, Eleni Dimou, Ioannis Lentzas, Dimitrios Levisianou, Afroditi Gova, Andreas Melidonis

**Affiliations:** 1 Department of General Practice/Family Medicine, Health Centre of Vyronas, Athens, Greece; 2 Diabetes Center, Tzanion General Hospital, Piraeus, Greece; 3 3 korytsas str., 16231 Vyronas, Athens, Greece

## Abstract

**Background:**

Patients with diabetes already fulfill one diagnostic criterion for MS according to the existing classifications. Our aim was to identify one single clinical parameter, which could effectively predict the presence of MS in patients with type 2 diabetes.

**Methods:**

We studied all patients with type 2 diabetes who attended our Diabetes Outpatient Clinic during a three-month period. Waist circumference, blood pressure and serum lipids were measured. Establishment of MS diagnosis was based a) on National Cholesterol Education Program Adult Treatment Panel III (NCEP ATP III) criteria and b) on International Diabetes Federation (IDF) criteria. Receiver operating characteristic (ROC) analysis was applied in order to identify the clinical parameter with the highest predictive capability for MS. Among the 500 participating patients (231 males, 269 females), MS was diagnosed in 364 patients (72.8%) according to the NCEP ATP III criteria and in 408 patients (81.6%) according to the IDF criteria.

**Results:**

For the NCEP ATP III classification, serum triglycerides (in the overall population), waist and HDL (in female population) demonstrated the highest predictive capability for MS (AUCs:0.786, 0.805 and 0.801, respectively). For the IDF classification, no single parameter reached an AUC > 0.800 in the overall population. In females, HDL displayed a satisfactory predictive capability for MS with an AUC which was significantly higher than the one in males (0.785 vs. 0.676, respectively, p < 0.05).

**Conclusion:**

Elevated serum triglycerides strongly indicate the presence of MS in patients with type 2 diabetes. In female patients with type 2 diabetes, central obesity was the second stronger predictor of MS besides hypertriglyceridemia.

## Background

Patients with the metabolic syndrome (MS) are at increased risk of coronary heart disease and other cardiovascular diseases related to plaque buildups in artery walls (e.g., stroke and peripheral vascular disease) [1]. Moreover MS also increases mortality from both cardiovascular disease and all causes [2,3]. Patients with type 2 diabetes already fulfill one of the NCEP ATP III or IDF diagnostic criteria, while at least two more are required for the confirmation of NCEP ATP III/M.S diagnosis, and necessarily abdominal obesity plus one additional criterion for the IDF/MS diagnosis [4,5]. Several studies have investigated the role of each of the metabolic syndrome components in modifying cardiovascular risk in patients with type 2 diabetes [6].

Our study aimed in identifying a single parameter – of those used for diagnosing MS according to NCEP ATP III and IDF classifications – which could predict the presence of the syndrome in patients with type 2 diabetes.

## Research design and methods

We studied all patients with type 2 diabetes who attended our Diabetes Outpatient Clinic during a three-month period in 2004. Pregnant females were excluded from the study. Waist circumference, systolic (SBP) and diastolic (DBP) blood pressure were measured, body mass index (BMI) was estimated and blood samples were drawn for serum lipids measurement after a 12-hour fast. Establishment of MS diagnosis was based: a) on NCEP ATP III criteria [4] and b) on IDF criteria [5]. The study was approved by the ethical review committee of our hospital. All patients gave an informed consent.

According to the NCEP ATP III classification, MS is defined as the presence of three or more of the following criteria: 1) increased waist circumference (>102 cm in males, >88 cm in females), 2) elevated serum triglycerides (≥150 mg/dl), 3) low HDL cholesterol (<40 mg/dl in males, <50 mg/dl in females), 4) hypertension (SBP ≥130 mmHg and/or DBP ≥85 mmHg) or medical treatment of previously diagnosed hypertension and 5) impaired fasting glucose (≥110 mg/dl). According to the new IDF definition, diagnosis of MS requires *central obesity *(defined as waist circumference ≥94 cm for Europid men and ≥80 cm for Europid women, with ethnicity specific values for other groups) plus any two of the following: 1) elevated serum triglycerides (≥150 mg/dl) or specific treatment for this lipid abnormality, 2) low HDL cholesterol (<40 mg/dl in males, <50 mg/dl in females) or specific treatment for this lipid abnormality, 3) hypertension (SBP ≥130 mmHg and/or DBP ≥85 mmHg) or medical treatment for previously diagnosed hypertension and 4) high fasting plasma glucose (≥100 mg/dl) or previously diagnosed type 2 diabetes.

We created receiver operating characteristics (ROC) curves of serum triglycerides, HDL, SBP, DBP and waist circumference for the prediction of MS and we used the areas under the corresponding curves (area under the curve, AUC) to evaluate the predictive efficiency of each MS parameter. Statistical analysis was performed with SPSS 10.0 (1999, Chicago, IL). Statistical significance was set at a level of p < 0.05. Variables with AUCs≥0.800 were considered as having satisfactory predictive capability for the presence of MS. We used the algorithms of Hanley and McNeil for comparison of AUCs derived from different samples [7] or from the same sample [8]. The critical level for the z statistic was set at 1.96. We also tried different cutoffs in the selected variables and estimated the corresponding sensitivity (SENS), specificity (SPEC), positive predictive value (PPV) and negative predictive value (NPV). Serum HDL and waist circumference were analyzed according to gender, since the cutoffs used in the two definitions for these variables are different in males and females.

## Results

500 patients (231 males and 269 females) were enrolled in the study. Metabolic syndrome was diagnosed in 364 patients (72.8%) according to the NCEP ATP III criteria and in 408 patients (81.6%) according to the IDF criteria. The concordance between the two classifications was moderate (kappa = 0.640, p < 0.001). 10 (2.7%) of the 364 patients diagnosed with MS according to the NCEP ATP III classification did not fulfill the IDF criteria for MS, while 54 (13.2%) of the 408 patients diagnosed with MS according to the IDF classification did not fulfill the NCEP ATP III criteria for MS. After stratification by gender, 213 females (79.1%) and 151 males (65.4%) had MS according to the NCEP ATP III classification (p = 0.001), while 232 females (86.2%) and 176 males (76.2%) had MS according to the IDF classification (p = 0.004). Demographic and other characteristics of the patients with MS vs. those without MS according to the two classifications are shown in table 1.

**Table 1:** Demographic, clinical and laboratory data of the patients

**Variable**	**MS according to NCEP-ATP III criteria**			**MS according to IDF criteria**		
	
	**With MS (n = 364)**	**Without MS (n = 136)**	**p value**	**With MS (n = 419)**	**Without MS (n = 81)**	**p value**
Age (years)*	65.9 ± 10.0	62.0 ± 12.9	0.002	65.6 ± 10.2	61.6 ± 13.6	0.010
**Diabetes duration (years)**	12.7 ± 8.4	11.5 ± 8.5	0.166	12.5 ± 8.3	11.7 ± 8.9	0.389
**BMI (kg/m^2^)**	30.0 ± 5.2	26.7 ± 4.3	<0.001	29.9 ± 5.0	25.9 ± 4.8	<0.001
**Waist (cm) males**	108 ± 11	98 ± 9	<0.001	107 ± 10	95 ± 11	<0.001
**Waist (cm) females**	105 ± 9	89 ± 17	<0.001	104 ± 10	89 ± 20	<0.001
**Serum HDL (mg/d) males**	42 ± 10	51 ± 11	<0.001	44 ± 11	51 ± 12	<0.001
**Serum HDL (mg/dl) females**	49 ± 10	61 ± 10	<0.001	50 ± 11	61 ± 10	<0.001
**Serum triglycerides (mg/dl)**	172 ± 91	106 ± 50	<0.001	164 ± 90	111 ± 57	<0.001
**SBP (mmHg)**	134 ± 19	126 ± 14	<0.001	134 ± 19	123 ± 11	<0.001
**DBP (mmHg)**	79 ± 11	75 ± 8	<0.001	79 ± 11	74 ± 6	<0.001
**Glycosylated hemoglobin (%)**	7.1 ± 1.3	7.0 ± 1.3	0.137	7.1 ± 1.3	7.0 ± 1.3	0.416

For the NCEP ATP III classification, serum triglycerides in the overall population, and waist circumference and HDL in females had the highest predictive ability for MS (AUCs: 0.786, 0.805 and 0.801, respectively). After stratification by gender, the AUC for triglycerides differed significantly (0.843 in females vs. 0.744 in males, z = 2.47, p < 0.05). In younger patients (<50 years old), AUC for serum triglycerides was 0.811 [95% confidence intervals (CI) 0.685–0.939, p < 0.001]. In females, serum triglyceride level was a stronger predictor of MS than waist circumference (0.843 vs. 0.805, respectively), but the difference did not reach statistical significance (z = 0.82, p = NS).

We have calculated SENS, SPEC, PPV, and NPV at different cutoffs for triglycerides (in the overall population) and for waist and HDL in females, to assess the specificity for MS diagnosis according to the NCEP ATP III classification (Table 2). We aimed at cutoffs with satisfactory specificity (≥70%) for MS diagnosis [9], but with a reasonably acceptable sensitivity (>60%) as well. The estimated PPVs were high, while the NPVs were low, as expected from the high prevalence of the syndrome in our patient population. A cutoff of 120 mg/dl for serum triglycerides showed a satisfactory SPEC (70%), while a higher cutoff of 140 mg/dl had an even higher SPEC (88%). Serum HDL cutoffs of 55 and 50 mg/dl showed SPEC (66% and 93%, respectively). A waist cutoff of 98 cm in females showed 72% SPEC and a higher cutoff of 100 cm had similar SPEC (75%). In the majority (73.3%) of females with waist >98 cm, hypertension was the criterion which accomplished the MS diagnosis, while in nearly all females with HDL <50 mg/dl or triglycerides >150 mg/dl (97.3% and 98.0%, respectively), MS diagnosis was accomplished by the waist criterion.

**Table 2:** Triglyceride, HDL and waiste circumference cutoffs and respective sensitivity and specificity of MS diagnosis according to NCEP-ATP III and IDF classifications.

**Cutoffs**	**NCEP-ATP III**				**IDF**			
**TGC (mg/dl)**	**SENS (%)**	**SPEC (%)**	**PPV (%)**	**NPV (%)**	**SENS (%)**	**SPEC (%)**	**PPV (%)**	**NPV (%)**

**120**	70	70	85	47	67	66	89	31
**130**	66	77	88	46	60	71	90	29
**140**	61	88	92	46	55	81	93	29
**150**	53	92	95	42	47	100*	100*	21
**HDL (mg/dl) Females**	**SENS (%)**	**SPEC (%)**	**PPV (%)**	**NPV (%)**	**SENS (%)**	**SPEC (%)**	**PPV (%)**	**NPV (%)**
50	53	93	93	34	49	100*	100*	20
55	71	66	89	38	67	62	92	29
60	87	48	86	50	84	31	90	31
**Waist (cm) Females**	**SENS (%)**	**SPEC (%)**	**PPV (%)**	**NPV (%)**	**SENS (%)**	**SPEC (%)**	**PPV (%)**	**NPV (%)**
90	97	52	88	82				
95	83	67	90	55				
98	79	72	90	41	**			
100	71	75	90	41				
102	57	77	90	32				

*Patients fulfill three IDF criteria.

**No AUCs calculated, because central obesity is a prerequisite for MS diagnosis with IDF criteria.

For the IDF classification, in the overall population, no single parameter reached an AUC≥0.800. In females, serum HDL displayed the highest predictive capability for MS with an AUC which was significantly higher than the one in males (0.785 vs. 0.676, respectively, p < 0.05). However, HDL cutoffs above the suggested 50 mg/dl cutoff in females exhibited SPEC lower than 70% (table 3).

The AUC for triglycerides was higher in females compared to males (0.803 vs. 0.719), as with the NCEP ATP III classification, but the observed difference did not reach statistical significance. However, serum triglycerides above the 150 mg/dl cutoff in the overall population and HDL below the 50 mg/dl cutoff in females had 100% SPEC and 100% PPV, since patients with diabetes, central obesity (a prerequisite risk factor for IDF MS) plus one lipid criterion fulfill three IDF criteria.

## Discussion

Patients with type 2 diabetes have higher cardiovascular risk than non-diabetic subjects, independently of other risk factors such as smoking, hypertension or hypercholesterolemia [10]. Moreover, the prevalence of coronary heart disease in diabetic patients increases significantly with the addition of MS components [6].

Prevalence of MS in patients with type 2 diabetes is considerably high (70–92%), as reported by several studies using either NCEP ATP III or World Health Organization (WHO) criteria [6,11-15]. In our patient population, the estimated prevalence of MS was similarly high as expected (72.8% based on the NCEP ATP III criteria and 81.6% based on the IDF criteria).

The identification of patients with MS among patients with type 2 diabetes is of great importance, since this population carries a cluster of cardiovascular risk factors and should be urged to show major compliance to their therapeutic regimen. The recognition of a single parameter with high efficiency in predicting MS in patients with type 2 diabetes was the primary aim of our study. ROC curves are commonly adopted in order to determine the predictive ability of various methods or parameters used for the confirmation of a disease, a clinical characteristic or an outcome.

In the overall population, serum triglycerides had a satisfactory predictive ability for NCEP ATP III MS, while for the IDF classification, no single parameter reached an AUC≥0.800. Serum triglycerides in females displayed the highest AUCs in both classifications. The predictive ability of triglycerides for the NCEP ATP III MS was higher in females compared to males, while for the IDF MS a similar difference did not reach statistical significance. Serum HDL proved to be useful in females for both classifications, but its predictive ability in males was not satisfactory. A serum HDL lower than 50 mg/dl in females with diabetes (HDL criterion fulfilled) predicted the presence of the whole syndrome with a specificity of 92%.

Furthermore, measurement of waist circumference although easily performed in every clinical setting (hospital, outpatient clinic, home), proved to be more useful in females than in males. A waist circumference of 98 cm in females with diabetes has been proved sufficient in predicting the presence of MS according to the NCEP ATP III classification.

## Limitations

There were several limitations in our study. The population sample was not so large as to yield statistically significant results for small differences between AUCs [7]. In particular, differences between clinical parameters in the same set of patients (e.g. serum triglycerides vs. waist circumference in females) should be tested with larger samples to minimize the possibility of type II error. In addition, our population sample was a selected one, since it consisted of patients who were regularly followed up in an outpatient clinic. It would be interesting to apply the same method in patients with recently diagnosed diabetes or in patients with incidentally diagnosed diabetes from a random population sample.

Nevertheless, the information retrieved from our study implies that a female with type 2 diabetes and central obesity most probably has the entire MS according to the NCEP ATP III classification.

## Conclusion

Elevated serum triglyceride level was the strongest single predictor which effectively indicated the presence of MS in both male and female patients with type 2 diabetes according to the NCEP ATP III classification. In female patients with type 2 diabetes, central obesity as implied by an increased waist circumference was the second stronger predictor of MS after hypertriglyceridemia.
